# Thoracoscopic versus open repair of CDH in cardiovascular stable neonates

**DOI:** 10.1007/s00464-015-4560-8

**Published:** 2015-10-21

**Authors:** Sophie Costerus, Katrin Zahn, Kees van de Ven, John Vlot, Lucas Wessel, Rene Wijnen

**Affiliations:** Department of Pediatric Surgery, Erasmus MC, Sophia Children’s Hospital, University Medical Centre, P.O. Box 2060, Dr. Molewaterplein 60/Room Sk-1262, 3000 CB Rotterdam, The Netherlands; Department of Pediatric Surgery, Mannheim University Medical Center, Mannheim, Germany

**Keywords:** Diaphragmatic hernia, Congenital, Minimal access surgery, Open repair, Recurrences, Neonates

## Abstract

**Background:**

Thoracoscopic surgery is an increasingly popular surgical technique to repair congenital diaphragmatic hernia (CDH). However, acidosis during surgery and the higher recurrence rate are considerable risk factors. The aim of this retrospective study is to compare the outcome of open versus thoracoscopic repair of the diaphragm in neonates with CDH with the same degree of cardiovascular and pulmonary illness who meet the criteria for thoracoscopic repair.

**Methods:**

Retrospective analysis of all patients of two large national reference centers for CDH born in the years 2008 through 2012, and meeting the criteria for surgical repair on cardiopulmonary and physiological criteria according to the CDH EURO consortium consensus and meeting the criteria for thoracoscopic repair according to the review by Vijfhuize et al. The surgical technical aspects were comparable in both centers.

**Results:**

108 patients were included, of whom 75 underwent thoracoscopic repair and 34 underwent open repair. The gestational age and lung-to-head ratio were significantly lower and stay on the ICU significantly longer in the open-repair group. The operation time was longer (178 vs. 150 min, *p* = .012) and the recurrence rate higher (18.9 vs. 5.9 %, *p* = .036) in the thoracoscopic-repair group. The arterial pH, pO_2_, pCO_2_ and base excess before and after thoracoscopic repair were all significantly different.

**Conclusion:**

After critical selection for thoracoscopic repair of left-sided CDH based on the patient’s preoperative condition, the outcomes of open repair were almost identical to those of thoracoscopic repair. A notable exception is the recurrence rate, which was significantly higher in the thoracoscopic-repair group. For the time being, thoracoscopic primary closure seems a safe and effective procedure, but efficacy of thoracoscopic patch repair has not been established.

Over the last decades, survival of neonates operated on for congenital diaphragmatic hernia (CDH) has improved, but management of the condition is still a challenge for pediatricians and pediatric surgeons alike [1, 2]. The prognosis is mainly determined by the degrees of pulmonary hypertension, pulmonary hypoplasia and abnormal morphology of the pulmonary vasculature [3–5].

Nowadays, only few centers perform thoracoscopic repair if the patient is cardiopulmonary stable. The criteria of stability still differ and have been published in several retrospective studies, but were never investigated prospectively [6].

Early studies reported higher recurrence rates after thoracoscopic repair; i.e. 5–25 versus 0–11 % after open repair. This was explained by the effect of the so-called learning curve. However, more recent studies still do not report lower recurrence rates after thoracoscopic repair [6]. Also the reported conversion rate of thoracoscopic surgery to open surgery varies (3.4–75.0 %), dependent on both technical and ventilatory problems. In these retrospective studies the open approach was associated with higher mortality, possibly due to a higher rate of comorbidities in neonates with large diaphragmatic defects and case selection bias.

All previous studies are retrospective and non-randomized. In these studies the differences in patient characteristics make it hard to compare outcomes of the two types of surgery. A pilot study reported that outcome of thoracoscopic surgery may be more detrimental due to the long-term consequences of hypercapnia and severe acidosis during surgery [7]. In an attempt to better define the risk of recurrence we conducted this 5-year retrospective study in patients with the same degree of cardiovascular and pulmonary illness. Comparability of the two groups allowed studying the effect of type of surgery on ventilation settings, length of stay at the pediatric intensive care unit (PICU) and the arterial blood gas changes.

## Methods

Since January 2008 CDH patients of the Rotterdam Group and the Mannheim Group, both tertiary centers, are being treated according to the CDH-EURO consortium recommendations [4]. This created a standard of care for all of the patients and recommendation for the timing of surgical repair. The surgeons who performed the thoracoscopic CDH surgery, 2 in Mannheim and 4 in Rotterdam, all had more than 2 years experience before the start of this study in 2008 and had been specifically trained for MAS in Pediatric Surgery.

**Fig. 1:**
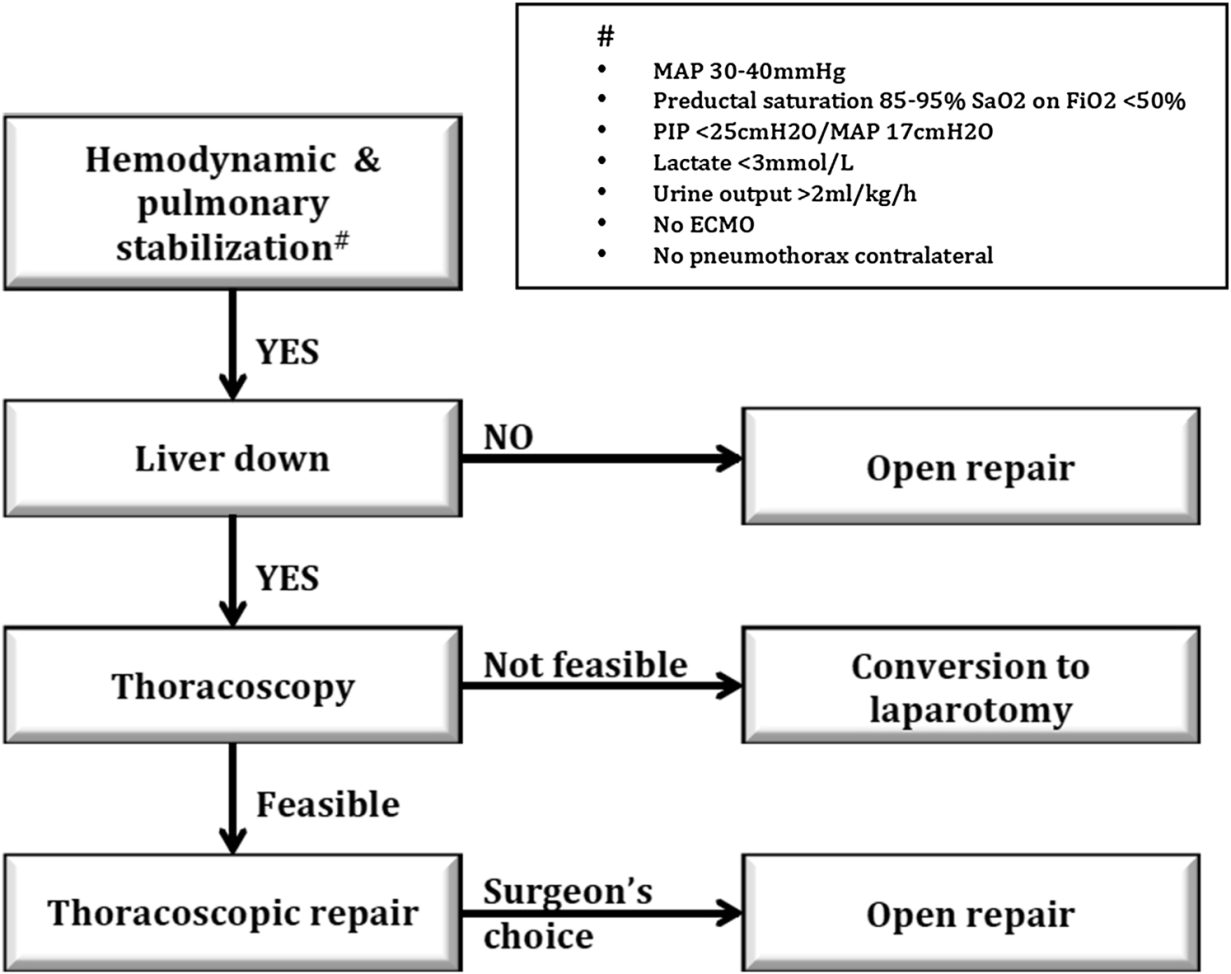
Decision tree for type of surgery

In a previous open review, we provided a decision tree for type of surgery (Fig. 1) [6]. The recommendations and the decision tree formed the selection criteria for thoracoscopic repair. A shared database was created with details of patients of two large pediatric surgery centers and who met the criteria for thoracoscopic repair in the period January 2008 through December 2012. The choice of surgery was based on surgeon’s preferences and logistic possibilities. This way it was possible to compare thoracoscopic repair and open repair without the bias of different patient characteristics, thus reducing case selection bias.

Included in this study were inborn CDH patients with a left-sided congenital diaphragmatic hernia (type Bochdalek) who had been operated upon within 30 days after birth. The diagnosis had been established either antenatally or postnatally. Data on gender, birth weight, gestational age, Apgar scores, prenatal diagnosis, lung-to-head ratio (LHR), liver position, need of ECMO and associated anomalies were obtained from medical records and entered in the shared database. Technical specifications of both types of surgeries were registered, i.e. suture material and patch material.

The following exclusion criteria applied: right-sided or bilateral diaphragmatic hernia, ECMO-treatment before surgery, HFO during surgery, intrathoracic liver position at preoperative ultrasound screening, and major cardiac anomalies.

The primary outcome was the number of recurrences within 1 year after surgery. The secondary outcomes were duration of surgery, postoperative number of days on ventilator, pCO_2_ and pH levels pre- and postoperatively, length of stay in the ICU and total hospital stay, duration until full enteral feeds and survival.

### Statistical analysis

Descriptive and non-paired *t* test statistics, as well as Mann–Whitney *U* tests, were performed with the use of SPSS 22 (SPSS Inc., Chicago, IL). The Fisher exact test was used for analysis of contingency tables. All data are presented as median. A *p* value <.05 was considered statistically significant.

## Results

The thoracoscopic group included 75 patients; the open group 34. Characteristics are shown in Table 1.

**Table 1:** Demographic characteristics

	Thoracoscopic group			Open group			*P* value
	25 %	Median	75 %	25 %	Median	75 %	
Number of patients		75			34		
Female (*n*)		34			18		.592
Gestational age (weeks)	37.90	38.28	38.57	36.86	38.00	38.42	.043
Birth weight (g)	2915	3045	3313	2538	2950	3180	.066
Apgar 1 min	5.5	7	8	5	7	7	.147
Apgar 5 min	8	8	9	8	8	8.25	.077
Apgar 10 min	8	9	9	8	8	9	.081
Lung-to-head ratio	1.8	1.9	2.4	1.3	1.7	2	.024

Demographic characteristics of the thoracoscopic and open surgery group. The data comparisons are shown in median with the 25 and 75 percentiles

The demographic characteristics of the two groups (see Table 1) did not significantly differ except for gestational age and lung-to-head ratio (LHR). The neonates in the open-repair group (OG) also had a lower median birth weight (2538 g, range 1140–3660) than the neonates in the thoracoscopic-repair group (TG) (2915 g, range 2340–3800 g), although this difference was not statistically significant.

The range of the lung-to-head ratio (LHR) in the TG was 0.90–4.90 and in the OG 1.00–3.00. Two neonates in the TG had a LHR under 1, three between 1–1.4, and 42 above 1.4. For the 28 other neonates in the TG the LHR had not been registered. None of the neonates in the OG had a LHR under 1, five between 1–1.4, and 12 above 1.4. For the other 17 neonates in this group the LHRs had not been registered.

At the preoperative screening the liver was assumed to be intra-abdominal in all neonates. During surgery it appeared that the left liver lobe was in the chest in 8.2 % of the TG and 23.5 % of the OG. In four cases, two in the TG and two in the OG, the position of the liver was not reported in the operation journal.

Pre-operative characteristics (Table 2) did not differ significantly between groups, except the ventilation settings (Table 3).

**Table 2:** Pre-operative characteristics

	Thoracoscopic group (*n* = 75)			Open group (*n* = 34)			*p* value
	25 %	Median	75 %	25 %	Median	75 %	
Heart rate	121	133	147	130	140	150	.418
Mean blood pressure	42.0	48.0	54.0	42.5	47.5	51.0	.776
pH	7.32	7.37	7.42	7.30	7.38	7.43	.826
pO_2_ (kPa)	11.93	15.48	20.01	12.66	17.34	21.21	.312
pCO_2 _(kPa)	4.75	5.54	6.53	5.05	5.85	6.50	.343
BE	−2.4	−1.0	2.0	−3.2	−1.2	2.6	.605
Highest pCO_2_ (kPa)	6.24	7.02	8.90	6.33	7.45	8.96	.749
Lowest pCO_2_ (kPa)	3.20	3.60	4.00	3.42	3.70	3.92	.758
Lactate (mmol/l)	0.8	1.1	1.4	1.0	1.2	1.5	.224
Urine output (ml/2 h)	16	25	35	16	23	34	.936

Comparison of preoperative characteristics in median with 25 and 75 percentiles. The data are the latest preoperative characteristics. Blood gas samples are preductal. Highest and lowest pCO_2_ are between admission PICU and surgical repair

**Table 3:** Pre-operative ventilation settings

	TG	OG	*P* value
No ventilation (*n*)	1.4 % (1)	0 % (0)	.045
CMV (*n*)	58.1 % (43)	79.4 % (27)	
HFO (*n*)	24.3 % (18)	5.9 % (2)	
PEEP (cm H_2_O)	4.50	3.50	.0001
FiO_2_ (%)	40	45	.050
NO (*n*)	5.4 % (4)	17.6 % (6)	.068

Comparison of preoperative ventilation settings

*TG* Thoracoscopic group, *OG* open group

The preoperative positive end expiratory pressure (PEEP) was significantly higher in the TG (range 3–7 mmHg vs. 2.5–6 mmHg in the OG). The fraction of inspired oxygen (FiO_2_) was higher in the OG, with a large range of 22–75 versus 21–60 % in the TG.

The duration of the total operation, including anesthesiological procedures, was significantly longer in the TG (*p* = .017). Large ranges were found: 58′–351′ in the TG, and 70′–244′ in the OG (Table 4). OG patients stayed longer in the ICU than did TG patients. The median difference was 5 days with a mean difference of 10 days.

**Table 4:** Pre- and postoperative characteristics

	Thoracoscopic group (*n* = 75)			Open group (*n* = 34)			*p* value
	25 %	Median	75 %	25 %	Median	75 %	25 %
Age at repair (days)	3	3	4	3	3	5.5	.525
Duration of operation (min)	143	178	219	114	150	192	.012
Primary surgical repair % (*n*)		41.3 % (31)			32.4 % (11)		.634
Patch repair % (*n*)		58.7 % (44)			67.6 % (23)		.184
Conversion % (*n*)		20.3 % (15)					
Properative liver (lobe) intrathoracic % (*n*)		8.2 % (6)			23.5 % (8)		.069
Extubation (days)	7	10	14	7	12	20	.265
Stay ICU (days)	11.0	16.5	24.0	14.5	21.0	35.0	.027
Recurrences % (*n*)		18.9 % (14)			5.9 % (2)		.036
Survival (%)		100 %			100 %		1

Comparison of the thoracoscopic and open surgery group in median, 25 and 75 percentiles

The recurrence rate in the TG was 9 out of 34 (26.5 %) in the Rotterdam Group and 5 out of 41 (12.2 %) in the Mannheim Group (*p* = .143), of which 6 and 4, respectively, concerned patch repair (*p* = 1.0). Both recurrences in the OG (5.9 %) had been previously repaired with a patch. Survival in both groups was 100 %.

The Rotterdam Group always uses Ethibond or Mersilene, both in open and thoracoscopic surgery. The Mannheim Group used Ethibond in primary closure (open and thoracoscopic) and thoracoscopic patch repair; only in open surgery with patch repair they used Tycron. All sutures are polyester (Table 5). All patients in both centers who needed patchclosure, GORE-TEX^®^ soft tissue patches were used.

**Table 5:** Technical specifications

	Rotterdam group	Mannheim group
Primary closure open surgery	Ethibond/Mersilene	Ethibond
Primary closure MAS	Ethibond/Mersilene	Ethibond
Patch closure open surgery	Mersilene	Tycron
Patch closure MAS	Ethibond/Mersilene	Ethibond
Knotting primary closure	Intracorporeal	Intracorporeal
Knotting patch closure	Intracorporeal	Intracorporeal

Comparison of the surgical and technical specifications between the Rotterdam and the Mannheim Group

Small, not clinically relevant changes were found in the comparison of the arterial blood gases before and after surgery. In the TG, the median pH decreased from 7.37 to 7.31; the median of the pCO_2_ increased from 5.54 to 5.93; and the median of the base excess decreased from −1.0 to −2.0. Nevertheless, as shown with the paired differences test, preoperative values for the pH, base excess, pCO_2_ and pO_2_ were significantly different from the corresponding postoperative values in the TG. In the OG, only base excess and pO_2_ were significantly different before and after surgery.

## Discussion

The traditional surgical management of CDH consists of repair through laparotomy. In the last decade, however, minimal access surgery (MAS) has gained wider popularity [6, 8].

Both are centers of expertise with respect to congenital diaphragmatic hernia neonates and pediatric surgery. Together they treat approximately 80 CDH neonates per year. The Mannheim Group performed their first MAS in 1993 and performed their first thoracoscopic surgical repair of a CDH neonate is 2008. The Rotterdam Group performed their first MAS in 1998 and operated thoracoscopically on the first CDH neonate in 2006.

Thoracoscopic repair for CDH is potentially associated with fewer postoperative ventilator days and possibly less use of analgesics [9, 10]. On the other hand, the artificial pneumoperitoneum (or pneumothorax) needed for MAS negatively affects hemodynamics [11–17].

Multiple studies show a higher recurrence rate associated with thoracoscopic repair, as is also found in the present study, due to learning curve, limited workspace and the use of a patch [6]. Furthermore the surgical difference between open and thoracoscopic repair is that the rim of diaphragm is mostly adhered to the dorsal pleuroperitoneal canal and preparation from thoracic side is challenging. From the abdominal route preparation is much easier and more obvious, while from thoracic route these structures are harder to find.

A recent prospective multicenter study showed a lower recurrence rate after open repair with patch than after MAS repair with patch during the first hospital stay [18]. In that study laparoscopy and thoracoscopy were clustered in one MAS group and the patient characteristics were not comparable between the open group and the MAS group. However, the high recurrence rate for the MAS group might be compensated for by the associated benefits mentioned above and by shorter ICU stay.

In the TG, recurrence rate in Rotterdam was 26.5 versus 12.2 % in Mannheim. To explain this discrepancy we looked at possible technical differences between both centers. In both centers, however, the surgery was performed only by a small group of surgeons with several years’ experience in MAS in neonates and large experience in CDH surgery. Moreover, the same type of sutures was used. Thus, the discrepancy may be due to the type of patch used. Loff et al. [19] from Mannheim showed reduced recurrence rates in open repair with a Dual Mesh cone-shaped patch. In the thoracoscopic repair with patch they also used the Dual Mesh cone-shaped patch, in contrast to the Rotterdam group. Furthermore, the conventional open patch repair is performed using a patch of the size of the defect or with an overlapping border of 1 cm circumferentially and sutured with interrupted non-absorbable material to the rim of the diaphragm [19]. Thoracoscopically the overlapping border is possibly smaller and the patch is sutured at the thoracic side instead of the abdominal side as in open surgery. The advantages of the cone-shaped patch are an increased abdominal capacity and reduction redundant chest capacity, thereby allowing normal physiological position of the abdominal organs, which in turn prevents gastroesophageal reflux and causes fewer recurrences because of separate fixation of the overlapping border of the cone and less abdominal pressure on the patch [19].

Several articles describe selection criteria for thoracoscopic repair [3, 6, 8, 9, 20, 21]. The cardiovascular criteria are almost the same in these overviews, i.e. no clinical signs of persistent pulmonary hypertension (PPHN), no need for inhaled nitric oxide (iNO) during surgery, and no need for ECMO. All agree that the patient has to be respiratory stable, but the definition of respiratory stable differs for PIP, PEEP, FiO_2_ and the pulse oxmeter oxygen saturation (SpO_2_). The ventilation criteria as recommended by the CDH consortium are a PEEP of 2–5 cm and in addition FiO_2_ < 50 % with a SpO_2_ between 85 and 95 %, so that both open and thoracoscopic repair are possible. These recommendations are based on nonanalytic studies, case reports, or expert opinions [4].

In the present study, only the preoperative differences in PEEP (4.5 vs. 3.5) between the TG and the OG suggest cardiopulmonary differences between the two groups. Two neonates in the TG had a LHR < 1.0 versus none in the OG, but data on the observed versus expected LHR were not available. Ten patients, 4 in the TG and 6 in the OG, received a low maintenance dose of iNO before surgery.

Patients after open repair stayed longer in the ICU than did patients after thoracoscopic repair. This may suggest that the former group had a worse pulmonary condition, but the median LHR in both groups was >1.4 and the ranges of the PEEP and FiO_2_ were not suggestive of worse condition. Still, the open repaired diaphragm defects, especially if patch closure was necessary, may suggest a bigger defect.

The artificial pneumothorax needed for thoracoscopic surgery creates acidosis due to hypercapnia by CO_2_ insufflation. This, in combination with higher intra-abdominal pressure, is believed to be related to deficient microcirculation [13, 16, 22, 23]. Splanchnic redistribution of blood flow with altered renal and hepatic function has been demonstrated. Also, testicular damage and reduced strength of (colonic) anastomoses have been reported as negative side effects of pneumoperitoneum [24, 25]. Nevertheless, patients with hypercapnia show global hyperperfusion of the cerebral blood flow [26]. The cerebral blood flow and volume depend on the ability of the cerebral arteries to respond to changes in the partial pressure of arterial CO_2_ [27].

Bishay et al. [7] recently proved severe arterial blood gas changes during thoracoscopic repair of CDH, but this finding was based on only 5 patients. We also showed a significant difference in pH and pCO_2_ values before and after thoracoscopic repair. However, these differences were small and deemed of limited clinical relevance. Due to the acidosis and hypercapnia, high-frequency oscillatory ventilation (HFOV) during neonatal thoracoscopic repair has gained attention in recent years. Mortellaro et al. [28] showed that this allowed good intraoperative exposure in correction of esophageal atresia and CDH, while allowing excellent oxygenation and elimination of carbon dioxide to prevent acidosis. Whether this ventilation technique can truly prevent the changes in acid–base balance associated with capnopneumothorax has yet to be established.

For this study, a selection of left-sided CDH patients meeting the criteria for thoracoscopic repair was made based on their preoperative condition. Some patients who met these criteria underwent open repair. These patients were, as a group, comparable to patients operated on thoracoscopically. The difference in overall recurrence rate that was found cannot be explained by the patient characteristics. Possible explanations are the position of the patch (abdominal vs. thoracic side of the defect) and the level of security with which the patch is sutured because the suture material, the knot tying and the patch material were the same. The only difference between the centers is the use of a cone-shaped patch as mentioned above. But still patchrepair in thoracoscopic surgery is more challenging and many centers convert to open surgery when a patch is needed for closing the diaphragm and only primary closure is performed thoracoscopically.

In both the TG and the OG the pO_2_ and the base excess values changed significantly from before to after surgery. In the TG, the pH and the pCO_2_ changed significantly as well. This is probably the result of CO_2_ insufflation during thoracoscopic surgery.

In addition, the overall time until recovery after surgery was longer for the OG. This does not seem to be due to a higher degree of pulmonary hypoplasia in this group. Perhaps the increased surgical trauma of open repair or maybe inadvertent selection bias may be responsible for this.

The effects of acidosis and hypercapnia on the (long-term) neurological development of the thoracoscopically repaired CDH patients are unknown. Thus there is a strong need for a well-designed study with state-of-the art neurological monitoring during thoracoscopic surgery but also for structured long-term follow up, looking at psychomotor development after this type of surgery. The higher recurrence rate in the thoracoscopically repaired group needs further attention, specifically when a patch is used. For the time being, thoracoscopic primary closure seems a safe and effective procedure, but efficacy of thoracoscopic patch repair has not been established.

## Abbreviations

CDH: Congenital diaphragmatic hernia
MAS: Minimal access surgery
ECMO: Extra corporeal membrane oxygenation
LHR: Lung-to-head ratio
PEEP: Positive end expiratory pressure
PIP: Peak inspiratory pressure
CMV: Conventional mechanical ventilation
HFOV: High-frequency oscillatory ventilation
Mean BP: Mean arterial blood pressure
PICU: Pediatric intensive care unit
NICU: Neonatal intensive care unit
PPHN: Persistent pulmonary hypertension
SEM: Standard error of the mean
LOS: Length of stay
TG: Thoracoscopically repaired group
OG: Open-repaired group
FiO_2_: Fraction inspired oxygen
SpO_2_: Pulse oximeter oxygen saturation
iNO: Inhaled nitric oxide
QLI: Quantitative lung index
MRI: Magnetic resonance imaging

## References

[1] Downard CD, Jaksic T, Garza JJ, Dzakovic A, Nemes L, Jennings RW, Wilson JM (2003). Analysis of an improved survival rate for congenital diaphragmatic hernia. J Pediatr Surg.

[2] Lally KP, Lasky RE, Lally PA, Bagolan P, Davis CF, Frenckner BP, Hirschl RM, Langham MR, Buchmiller TL, Usui N, Tibboel D, Wilson JM, Congenital Diaphragmatic Hernia Study G (2013). Standardized reporting for congenital diaphragmatic hernia—an international consensus. J Pediatr Surg.

[3] Gomes Ferreira C, Kuhn P, Lacreuse I, Kasleas C, Philippe P, Podevin G, Bonnard A, Lopez M, De Lagausie P, Petit T, Lardy H, Becmeur F (2013). Congenital diaphragmatic hernia: an evaluation of risk factors for failure of thoracoscopic primary repair in neonates. J Pediatr Surg.

[4] Reiss I, Schaible T, van den Hout L, Capolupo I, Allegaert K, van Heijst A, Gorett Silva M, Greenough A, Tibboel D, Consortium CE (2010). Standardized postnatal management of infants with congenital diaphragmatic hernia in Europe: the CDH EURO Consortium consensus. Neonatology.

[5] Tovar JA (2012). Congenital diaphragmatic hernia. Orphanet J Rare Dis.

[6] Vijfhuize S, Deden AC, Costerus SA, Sloots CE, Wijnen RM (2012). Minimal access surgery for repair of congenital diaphragmatic hernia: is it advantageous? An open review. Eur J Pediatric Surg.

[7] Bishay M, Giacomello L, Retrosi G, Thyoka M, Garriboli M, Brierley J, Harding L, Scuplak S, Cross KM, Curry JI, Kiely EM, De Coppi P, Eaton S, Pierro A (2013). Hypercapnia and acidosis during open and thoracoscopic repair of congenital diaphragmatic hernia and esophageal atresia: results of a pilot randomized controlled trial. Ann Surg.

[8] Gourlay DM, Cassidy LD, Sato TT, Lal DR, Arca MJ (2009). Beyond feasibility: a comparison of newborns undergoing thoracoscopic and open repair of congenital diaphragmatic hernias. J Pediatr Surg.

[9] Lao OB, Crouthamel MR, Goldin AB, Sawin RS, Waldhausen JH, Kim SS (2010). Thoracoscopic repair of congenital diaphragmatic hernia in infancy. J Laparoendosc Adv Surg Tech Part A.

[10] Ceelie I, van Dijk M, Bax NM, de Wildt SN, Tibboel D (2011). Does minimal access major surgery in the newborn hurt less? An evaluation of cumulative opioid doses. Eur J Pain.

[11] Blobner M, Bogdanski R, Kochs E, Henke J, Findeis A, Jelen-Esselborn S (1998). Effects of intraabdominally insufflated carbon dioxide and elevated intraabdominal pressure on splanchnic circulation: an experimental study in pigs. Anesthesiology.

[12] Caldwell CB, Ricotta JJ (1987). Changes in visceral blood flow with elevated intraabdominal pressure. J Surg Res.

[13] Gutt CN, Oniu T, Mehrabi A, Schemmer P, Kashfi A, Kraus T, Buchler MW (2004). Circulatory and respiratory complications of carbon dioxide insufflation. Dig Surg.

[14] Ishizaki Y, Bandai Y, Shimomura K, Abe H, Ohtomo Y, Idezuki Y (1993). Changes in splanchnic blood flow and cardiovascular effects following peritoneal insufflation of carbon dioxide. Surg Endosc.

[15] Jakimowicz J, Stultiens G, Smulders F (1998). Laparoscopic insufflation of the abdomen reduces portal venous flow. Surg Endosc.

[16] Nickkholgh A, Barro-Bejarano M, Liang R, Zorn M, Mehrabi A, Gebhard MM, Buchler MW, Gutt CN, Schemmer P (2008). Signs of reperfusion injury following CO_2_ pneumoperitoneum: an in vivo microscopy study. Surg Endosc.

[17] Ure BM, Suempelmann R, Metzelder MM, Kuebler J (2007). Physiological responses to endoscopic surgery in children. Semin Pediatr Surg.

[18] Tsao K, Lally PA, Lally KP, Congenital Diaphragmatic Hernia Study G (2011). Minimally invasive repair of congenital diaphragmatic hernia. J Pediatr Surg.

[19] Loff S, Wirth H, Jester I, Hosie S, Wollmann C, Schaible T, Ataman O, Waag KL (2005). Implantation of a cone-shaped double-fixed patch increases abdominal space and prevents recurrence of large defects in congenital diaphragmatic hernia. J Pediatr Surg.

[20] Yang EY, Allmendinger N, Johnson SM, Chen C, Wilson JM, Fishman SJ (2005). Neonatal thoracoscopic repair of congenital diaphragmatic hernia: selection criteria for successful outcome. J Pediatr Surg.

[21] Szavay PO, Obermayr F, Maas C, Luenig H, Blumenstock G, Fuchs J (2012). Perioperative outcome of patients with congenital diaphragmatic hernia undergoing open versus minimally invasive surgery. J Laparoendosc Adv Surg Tech Part A.

[22] Ben-Haim M, Rosenthal RJ (1999). Causes of arterial hypertension and splachnic ischemia during acute elevations in intra-abdominal pressure with CO_2_ pneumoperitoneum: a complex central nervous system mediated response. Int J Colorectal Dis.

[23] Schilling MK, Redaelli C, Krahenbuhl L, Signer C, Buchler MW (1997). Splanchnic microcirculatory changes during CO_2_ laparoscopy. J Am Coll Surg.

[24] Imamoglu M, Cay A, Unsal MA, Aydin S, Ozdemir O, Karahan C, Sari A, Sarihan H (2006). The effects of increased intraabdominal pressure on testicular blood flow, oxidative stress markers, and morphology. J Pediatr Surg.

[25] Polat C, Arikan Y, Vatansev C, Akbulut G, Yilmaz S, Dilek FH, Gokce O (2002). The effects of increased intraabdominal pressure on colonic anastomoses. Surg Endosc.

[26] Pollock JM, Deibler AR, Whitlow CT, Tan H, Kraft RA, Burdette JH, Maldjian JA (2009). Hypercapnia-induced cerebral hyperperfusion: an underrecognized clinical entity. AJNR Am J Neuroradiol.

[27] Madden JA (1993). The effect of carbon dioxide on cerebral arteries. Pharmacol Ther.

[28] Mortellaro VE, Fike FB, Adibe OO, Juang D, Aguayo P, Ostlie DJ, Holcomb GW, St Peter SD (2011). The use of high-frequency oscillating ventilation to facilitate stability during neonatal thoracoscopic operations. J Laparoendosc Adv Surg Tech Part A.

